# Supplementary material for: Venomix: a simple bioinformatic pipeline for identifying and characterizing toxin gene candidates from transcriptomic data
Source: PeerJ. 2018 Jul 31;6:e5361. doi: 10.7717/peerj.5361 (PMC6074769; doi:10.7717/peerj.5361)
Supplement: Supplemental Information 3 [file peerj-06-5361-s003.gz › FinalOutput_E-6/Kunitz-type_serine_protease_inhibitor_bitisilin-3_1/finaltree.pdf]

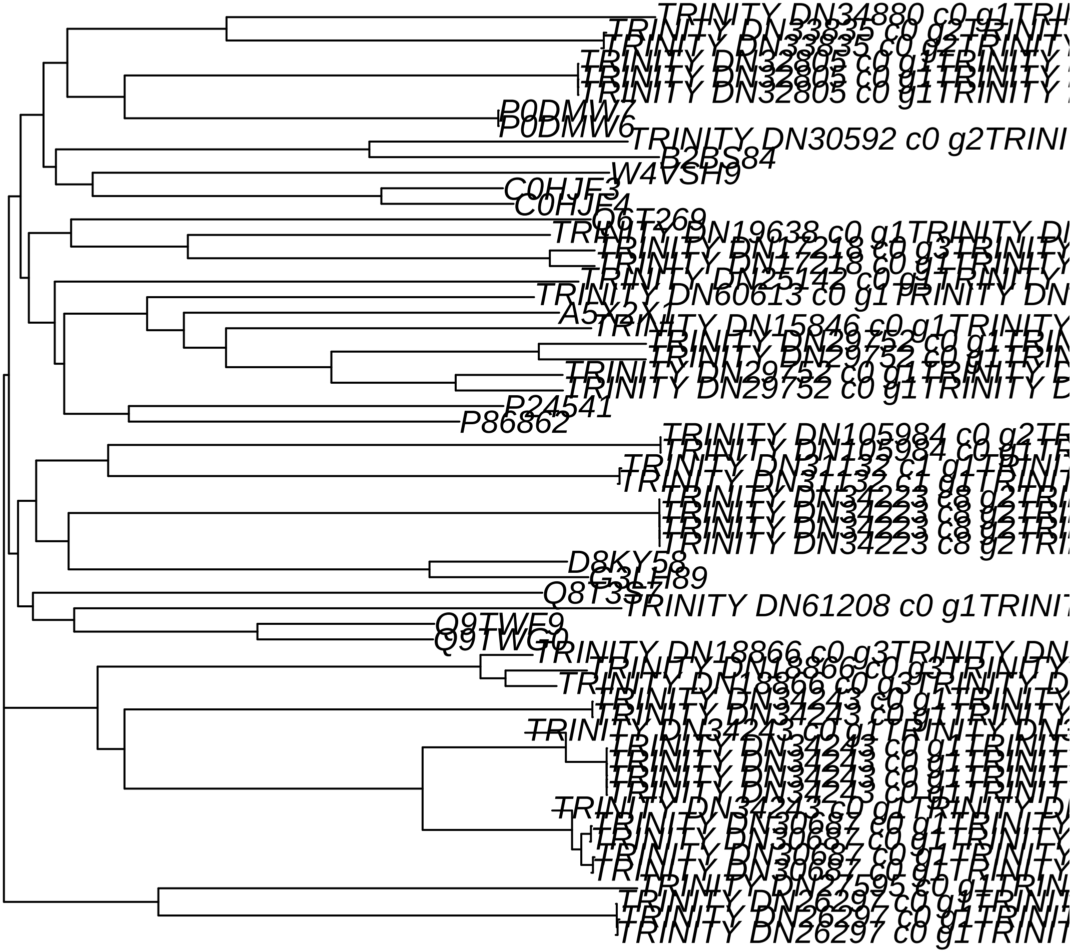
TRINITY DN34880 c0 g1TRIN
TRINITY DN33835 c0 g2TRINITY
TRINITY DN33835 c0 g2TRINITY
TRINITY DN32805 c0 g1TRINITY
TRINITY DN32805 c0 g1TRINITY
TRINITY DN32805 c0 g1TRINITY
P0DMW7
P0DMW6
TRINITY DN30592 c0 g2TRINI
B2BS84
W4VSH9
C0HJF3
C0HJF4
Q6T269
TRINITY DN19638 c0 g1TRINITY DI
TRINITY DN17218 c0 g3TRINITY
TRINITY DN17218 c0 g1TRINITY
TRINITY DN25142 c0 g1TRINITY
TRINITY DN60613 c0 g1TRINITY DN
A5X2X1
TRINITY DN15846 c0 g1TRINITY
TRINITY DN29752 c0 g1TRIN
TRINITY DN29752 c0 g1TRIN
TRINITY DN29752 c0 g1TRINITY
TRINITY DN29752 c0 g1TRINITY D
P24541
P86862
TRINITY DN105984 c0 g2TR
TRINITY DN105984 c0 g1TR
TRINITY DN31132 c1 g1TRINI
TRINITY DN31132 c1 g1TRINI
TRINITY DN34223 c8 g2TRI
TRINITY DN34223 c8 g2TRI
TRINITY DN34223 c8 g2TRI
TRINITY DN34223 c8 g2TRI
D8KY58
G3LH89
Q8T3S7
TRINITY DN61208 c0 g1TRINIT
Q9TWF9
Q9TWG0
TRINITY DN18866 c0 g3TRINITY DN
TRINITY DN18866 c0 g3TRINITY
TRINITY DN18866 c0 g3TRINITY D
TRINITY DN34243 c0 g1TRINITY
TRINITY DN34243 c0 g1TRINITY
TRINITY DN34243 c0 g1TRINITY DN3
TRINITY DN34243 c0 g1TRINIT
TRINITY DN34243 c0 g1TRINIT
TRINITY DN34243 c0 g1TRINIT
TRINITY DN34243 c0 g1TRINIT
TRINITY DN34243 c0 g1TRINITY DI
TRINITY DN30687 c0 g1TRINITY
TRINITY DN30687 c0 g1TRINITY
TRINITY DN30687 c0 g1TRINITY
TRINITY DN30687 c0 g1TRINITY
TRINITY DN27595 c0 g1TRIN
TRINITY DN26297 c0 g1TRINI
TRINITY DN26297 c0 g1TRINI
TRINITY DN26297 c0 g1TRINIT
